# Female XX sex chromosomes increase survival and extend lifespan in aging mice

**DOI:** 10.1111/acel.12871

**Published:** 2018-12-17

**Authors:** Emily J. Davis, Iryna Lobach, Dena B. Dubal

**Affiliations:** ^1^ Department of Neurology, Biomedical Sciences Graduate Program, and Weill Institute for Neurosciences University of California, San Francisco San Francisco California; ^2^ Department of Epidemiology and Biostatistics University of California, San Francisco San Francisco California

**Keywords:** aging, four core genotype, life-span studies, mortality, mouse models, sex differences, sex hormones

## Abstract

Female longevity is observed in humans and much of the animal kingdom, but its causes remain elusive. Using a genetic manipulation that generates XX and XY mice, each with either ovaries or testes, we show that the female XX sex chromosome complement increases survival during aging in male and female mice. In combination with ovaries, it also extends lifespan. Understanding causes of sex‐based differences in aging could lead to new pathways to counter age‐induced decline in both sexes.

## 1 INTRODUCTION

Women live longer than men around the world, regardless of culture or socioeconomic status (UnitedNations, 2015; Zarulli et al., 2018). Female longevity is also observed in the animal kingdom (Barrett & Richardson, 2011; Bronikowski et al., 2011; Clutton‐Brock & Isvaran, 2007) due to causes that may be extrinsic, intrinsic, or both. Extrinsic causes of sex difference in invertebrates can signal antagonistic survival strategies: female pheromones reduce male lifespan in *Drosophila* (Gendron et al., 2014), and male secretions shorten hermaphrodite lifespan in *C. elegans* (Maures et al., 2014). Intrinsic effects—operating within the organism—underlie longer life in organisms following removal of reproductive cells or organs in *C. elegans* hermaphrodites (Berman & Kenyon, 2006), male and female dogs (Hoffman, Creevy, & Promislow, 2013), and possibly men as suggested by a study of eunuchs (Min, Lee, & Park, 2012). Nonetheless, causes of intrinsic sex difference in lifespan remain largely unknown. The pervasive nature of female longevity in humans, even in early death during severe epidemics and famine (Zarulli et al., 2018), suggests a role for innate biology in the survival gap between the sexes. Here, we sought to identify intrinsic causes of female longevity in mammalian lifespan.

Sex chromosomes or gonads cause intrinsic sex differences in mammals, but whether they directly contribute to increased female lifespan is unknown in mammalian aging. To dissect these etiologies, we used four core genotypes (FCG) mice (Arnold, 2004). In mice and humans, the *Sry* gene normally resides on the Y chromosome and codes for a protein (testicular determining Y factor) that induces development of testes and perinatal masculinization. In FCG mice, *Sry* resides instead on an autosome, enabling inheritance of *Sry*—and thus male, testicular phenotype—with or without the Y chromosome.

**Figure 1:**
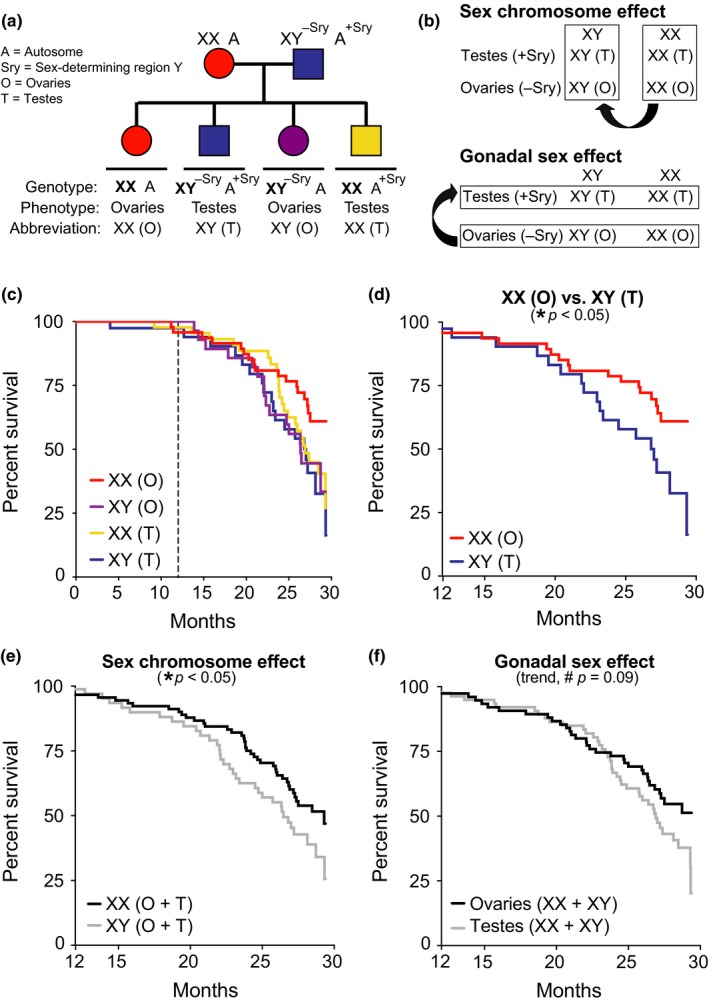
XX sex chromosomes contribute to female longevity. (a) Diagram of FCG model. XX females were crossed with XY males with the *Sry* on an autosome instead of the Y chromosome. (b) Strategy to identify causes of sexual dimorphism using the FCG model by testing main effect of sex chromosomes (top) and main effect of gonads (bottom). (c–f) Kaplan–Meier curves of FCG aging cohort (*n* = 261 mice): XX(O) *n* = 64, XY(T) *n* = 48, XX(T) *n* = 94, and XY(O) *n* = 55. (c) In all groups, survival was tracked until 30 months and statistical analyses were performed with left censoring prior to 12 months as indicated by dotted vertical line. (d) Stratified pairwise hazard model comparisons show that XX(O) mice exhibit less mortality than XY(T) mice (XX(O), HR = 0.45, CI = 0.23–0.88, *p = 0.02). Cox proportional hazard model analysis shows (e) main effect of sex chromosome complement (XX, HR = 0.60, CI = 0.37–0.96, *p = 0.03) and (f) trend in gonadal effect (ovaries, HR = 0.66, CI = 0.41–1.06, #*p* = 0.09). HR = hazard ratio and CI = confidence interval; HR < 1 is decreased mortality risk (statistical details in Supporting Information Tables S1 and S2)

The genetic manipulation of SRY generates XX and XY mice, each with either ovaries (O) or testes (T): XX(O), XX(T), XY(O), XY(T) (Figure 1a). Gonadal hormone levels in FCG mice with the same gonads are comparable, regardless of their sex chromosomes (Gatewood et al., 2006; McCullough et al., 2016). In FCG model mice, a sex difference with a main effect that statistically differs by genotype (XX vs. XY) is sex chromosome‐mediated; one that differs by phenotype (ovaries vs. testes) is gonadal sex‐mediated (Figure 1b). Examples of age‐relevant FCG mouse studies show that XX improves blood pressure regulation (Pessoa et al., 2015) and attenuates experimental brain injuries (Du et al., 2014; McCullough et al., 2016).

To explore sex‐based differences in lifespan, we generated and aged over 200 mice from the FCG model on a congenic C57BL/6J background and investigated aging‐dependent mortality from midlife to old age (12–30 months) (Figure 1c). We first examined whether mortality in “typical” females (XX,O) and males (XY,T) recapitulates the pattern of female longevity. Indeed, aging females (XX,O) lived longer than aging males (XY,T) (Figure 1d; Supporting Information Table S1).

We next measured main effects of sex chromosomes and gonads on survival in aging. XX mice with ovaries or testes lived longer than XY mice of either gonadal phenotype, indicating a main effect of sex chromosomes on lifespan (Figure 1e; Supporting Information Table S2). Mice with ovaries (XX & XY) tended to live longer than those with testes (XX & XY), suggesting a gonadal influence on lifespan (Figure 1f; Supporting Information Table S2). Collectively, these data indicate that the XX genotype increases survival in aging—and suggest a protective effect of ovaries.

**Figure 2:**
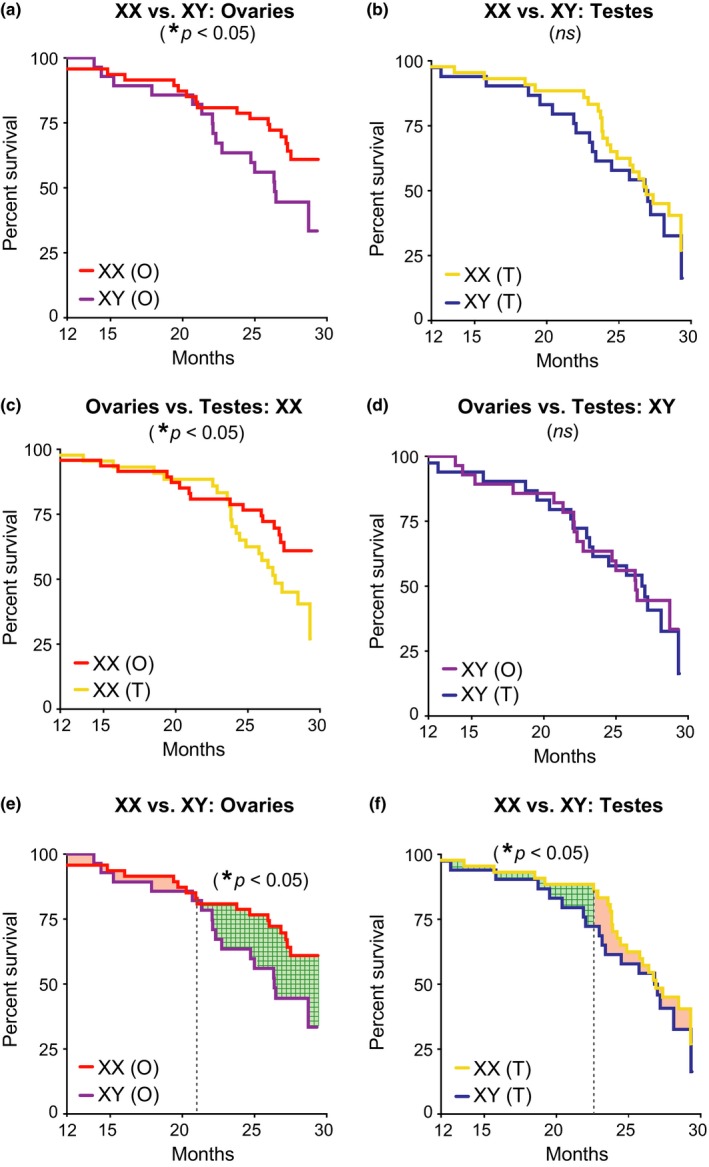
XX sex chromosomes extended lifespan in combination with ovaries and independently increased survival during aging. (a–f) Kaplan–Meier curves of FCG aging cohort (*n* = 261 mice): XX(O) *n* = 64, XY(T) *n* = 48, XX(T) *n* = 94, and XY(O) *n* = 55. (a) In mice with ovaries, XX decreased mortality compared to XY (XX, HR = 0.46, CI = 0.23–0.94, *p = 0.03). (b) In mice with testes, mortality tended to be higher overall and did not differ between XX and XY genotypes (XX, HR = 0.81, CI = 0.43–1.50, *p* = 0.53). (c) In XX mice, ovaries decreased mortality compared to testes (ovaries, HR = 0.51, CI = 0.26–0.99, *p = 0.05). (d) In XY mice, mortality was lower overall and did not differ between those with testes or ovaries (ovaries, HR = 0.96, CI = 0.48–1.90, *p* = 0.90). (e, f) XX increased survival during aging in mice with ovaries and testes, as determined by a grid search method that statistically identifies the point in time that curves change in relation to each other (indicated by dotted vertical line); differences in lifespan before and after that time point are shaded (significant differences = green grid pattern; no difference = shaded red). (e) In mice with ovaries, the relationship between XX and XY lifespan curves changed at 21 months with no difference before then (XX, HR = 0.52, *SE* = 0.64, *p* = 0.31) and significant difference afterward (XX, HR = 0.37, *SE* = 0.45, *p = 0.01). (f) In mice with testes, the relationship between XX and XY lifespan curves changed at 23 months with a significant difference before then (XX, HR = 0.36, *SE* = 0.60, *p  < 0.05) and no difference afterward (HR = −0.78, *SE* = 0.33, *p* = 0.23). HR = hazard ratio, CI = confidence interval, and *SE* = standard error; HR < 1 is decreased mortality risk (statistical details in Supporting Information Tables S3–S6)

To further understand benefits of femaleness on survival in aging, we directly compared the four groups of mice. In mice with ovaries, XX increased lifespan compared to XY (Figure 2a; Supporting Information Table S3). In mice with testes, mortality tended to be higher overall and did not differ between XX and XY genotypes (Figure 2b; Supporting Information Table S3). Ovaries increased lifespan in XX, but not XY mice (Figure 2c,d; Supporting Information Table S4). This suggests that female gonadal hormones, through organizational (long‐term) or activational (short‐term) effects, increase lifespan in the presence of a second X chromosome.

Since the XX genotype showed a main effect on overall survival, we next tested whether it increases resilience against death anytime during aging. We used the grid search method (Lerman, 1980) to determine the point in time when XX and XY lifespan curves change in relation to each other in mice with matching gonads. We then measured statistical differences between the two curves before and after that point to assess whether XX increases survival at any time in aging. In mice with ovaries, XX increased survival after 21 months (Figure 2e; Supporting Information Table S5). In mice with testes, XX also increased survival, but the benefit was earlier, prior to 23 months, and did not alter maximal lifespan (Figure 2f; Supporting Information Table S6). Thus, independent of maximal lifespan, the XX genotype increased survival during aging in both male and female mice, albeit at different times.

It is important to note that lifespan and its interventions in mice are influenced by strain, substrain, environment, diet, and factors yet unidentified (Austad & Fischer, 2016). Thus, the presence, extent, and direction of sex bias in lifespan can vary across mouse colonies, even among C57BL6 substrains. Future studies examining mixed genetic backgrounds across geographic sites will be valuable. Nonetheless, our data are clear and indicate that female sex derived from the XX sex chromosome complement, combined with ovarian gonad exposure, extended lifespan; furthermore, the XX genotype itself increased survival in aging male and female mice.

Whether the presence of a second X chromosome or the lack of a Y dictates genetic causes of this intrinsic female advantage remains to be determined. Further, how hormone signaling induces ovarian‐mediated survival in the presence of a second X chromosome deserves study. Major pathways underlying an XX‐ovarian interaction could include IGF1 signaling (Brooks & Garratt, 2017), telomeres (Barrett & Richardson, 2011), or mitochondrial functions (Gaignard et al., 2015).

Evolutionary pressure may lie upon increased survival and longer lifespan in females to ensure additional care and better fitness for generations of genetic offspring. Alternatively, more male death could benefit the next generation by reducing competition for resources and mates. The identification and modulation of intrinsic XX‐derived mechanisms of female advantage could open new pathways to modify and increase healthy aging in both sexes.

## AUTHOR CONTRIBUTIONS

E.J.D., I.L., and D.B.D. carried out experimental studies and analyses and wrote the manuscript. All authors discussed results and commented on the manuscript.
