# Supplementary material for: Female XX sex chromosomes increase survival and extend lifespan in aging mice
Source: Aging Cell. 2018 Dec 17;18(1):e12871. doi: 10.1111/acel.12871 (PMC6351820; doi:10.1111/acel.12871)
Supplement: Supplementary file 1 [file ACEL-18-e12871-s001.docx]

**SUPPORTING INFORMATION FOR:**

**Title:** Female XX sex chromosomes increase survival in aging and extend lifespan in aging mice

**Authors:** Emily J. Davis, Iryna Lobach, Dena B. Dubal

**Supporting Information**

- Experimental Procedures
- Supporting Tables S1-6
- References

**Experimental Procedures**

**Transgenic Mice.**

Mice for survival curve studies were on a congenic C57BL/6J background and kept on a 12-h light/dark cycle with *ad libitum* access to food and water. Mice were fed standard rodent chow (LabDiet PicoLab Rodent Diet 20 #5053). The standard housing group was five mice per cage based on gonadal sex. Cage bedding was changed once per week. Mice were genotyped using primers to detect the presence of the:

Y chromosome:

SSTY1 Forward primer: CTGGAGCTCTACAGTGATGA

SSTY1 Reverse primer: CAGTTACCAATCAACACATCAC

SRY gene:

SRY Forward primer: AGCCCTACAGCCACATGATA

SRY Reverse primer: GTCTTGCCTGTATGTGATGG

Autosomal control gene

MYO Forward primer: TTACGTCCATCGTGGACAGCAT

MYO Reverse primer: TGGGCTGGGTGTTAGTCTTAT

All animal studies were approved by the Institutional Animal Care and Use Committee of the University of California, San Francisco and conducted in compliance with NIH guidelines.

**Inclusion/Exclusion criteria for survival curves**

All mice included in this study were bred from the Fore Core Genotypes model (Arnold, 2004; Lovell-Badge & Robertson, 1990; Mahadevaiah et al., 1998). Mice used for breeding (XY,T) and mice that went missing (random genotypes) were excluded from the analyses. Survival was assessed following weaning at approximately 21 days up until the age indicated. Some mice were euthanized to adhere to the stringent criteria developed by UCSF LARC veterinarians to prevent suffering. Deaths were censored if they occurred for reasons unrelated to natural aging, including: fight wounds, prolapsed anus, eyes bulging, paraphimosis, and dehydration. Ages are represented by months and each month is defined as 30 days.

**Statistics**

Cox proportional hazards models were applied to estimate main effects of sex chromosome complement and gonads. Specific pairwise comparisons within sex chromosome complement and gonadal type were made using a proportional hazard model with stratification of genotype and phenotype. Prior to using Cox proportional hazard models, potential deviations from the proportional hazard assumption were examined in a model with time-varying coefficients; there was not sufficient evidence to support a time-varying effect of the variables.

A grid search method (Lerman, 1980) was used to test if there is a difference in the survival pattern between XX vs. XY in mice with ovaries and in mice with testes. The method estimates a point in time at which the relative survival pattern between two curves changes. The change point is the time when survival between two curves is different before that time and no longer different after that time; or vice versa. We then examined differences in the survival patterns before and after the determined time point with Cox proportional hazard models. Since we previously found that XX increases survival as a main effect, the hypothesis following the grid search method was formulated as one-sided to determine when in the lifespan XX increases survival compared to XY in mice with ovaries or testes.

**Supporting Tables**

| **Table S1. Stratified proportional hazard model shows XX(O) had significantly decreased mortality compared to the each of the other FCG experimental groups.** HR=Hazard Ratio estimate, risk of death, CI=Confidence Interval. References are XX and ovaries. Table summarizes results in **Fig. 1c,d**.   \|  \| **HR** \| **95% CI** \| **P value** \| **Significance** \| \| --- \| --- \| --- \| --- \| --- \| \| **XX(O) vs. XY(T)** \| 0.45 \| 0.23-0.88 \| 0.020 \| * \| \| **XX(O) vs. XX(T)** \| 0.51 \| 0.26-0.99 \| 0.046 \| * \| \| **XX(O) vs. XY(O)** \| 0.46 \| 0.23-0.94 \| 0.033 \| * \|   **Table S2. Cox proportional hazard model shows significant main effect of sex chromosome complement.** XX decreases mortality and ovaries tend to decrease mortality. HR=Hazard Ratio estimate, risk of death, CI=Confidence Interval. References are XX and ovaries. Table summarizes results in **Fig. 1e,f**. |
| --- | --- | --- | --- | --- | --- | --- | --- | --- | --- | --- | --- | --- | --- | --- | --- | --- | --- | --- | --- | --- |

| \|  \| **HR** \| **SE** \| **z** \| **95% CI** \| **P value** \| **Significance** \| \| --- \| --- \| --- \| --- \| --- \| --- \| --- \| \| **Main Effect:**  **XY vs. XX** \| 0.60 \| 0.14 \| -2.15 \| 0.37-0.96 \| 0.032 \| * \| \| **Main Effect:**  **Testes vs Ovaries** \| 0.66 \| 0.16 \| -1.72 \| 0.41-1.06 \| 0.086 \| # \| |
| --- | --- | --- | --- | --- | --- | --- | --- | --- | --- | --- | --- | --- | --- | --- | --- | --- | --- | --- | --- | --- | --- |

| **Table S3. Stratified proportional hazard model of sex chromosome effect within gonadal sex group.** XX sex chromosome complement decreased overall mortality in mice with ovaries but not in those with testes. HR=Hazard Ratio estimate, risk of death. CI=Confidence Interval. Reference is XX genotype. Table summarizes results in **Fig. 2a,b**. |
| --- |

| \|  \| **HR** \| **95% CI** \| **P value** \| **Significance** \| \| --- \| --- \| --- \| --- \| --- \| \| **XY vs. XX: Mice with Ovaries** \| 0.46 \| 0.23-0.94 \| 0.033 \| * \| \| **XY vs. XX: Mice with Testes** \| 0.81 \| 0.43-1.5 \| 0.53 \| n.s. \| |
| --- | --- | --- | --- | --- | --- | --- | --- | --- | --- | --- | --- | --- | --- | --- | --- |

| **Table S4. Stratified proportional hazard model of gonadal sex effect within sex chromosome group.** Ovaries decrease mortality in XX but not in XY mice. HR=Hazard Ratio estimate, risk of death. CI=Confidence Interval. Reference is Ovaries. Table summarizes results given in **Fig. 2c,d**. |
| --- |

| \|  \| **HR** \| **95% CI** \| **P value** \| **Significance** \| \| --- \| --- \| --- \| --- \| --- \| \| **Testes vs. Ovaries: Mice with XX Genotype** \| 0.51 \| 0.26-0.99 \| 0.046 \| * \| \| **Testes vs. Ovaries: Mice with XY Genotype** \| 0.96 \| 0.48-1.9 \| 0.900 \| n.s. \| |
| --- | --- | --- | --- | --- | --- | --- | --- | --- | --- | --- | --- | --- | --- | --- | --- |

**Table S5. Stratified proportional hazard model following grid search method (Lerman, 1980) in mice with ovaries.** XX decreases death during aging, compared to XY, after 21 months of age. HR=Hazard Ratio estimate, risk of death. SE=Standard Error. Reference is XX genotype. Table summarizes results in **Fig. 2e.**

| **Time interval** | **HR** | **SE** | **P-value** | **Significance** |
| --- | --- | --- | --- | --- |
| 12mo-21mo | 0.52 | 0.64 | 0.31 | n.s. |
| 21mo-30mo | 0.37 | 0.45 | 0.013 | * |

**Table S6. Stratified proportional hazard model following grid search method (Lerman, 1980) in mice with testes.** XX decreases early death during aging, compared to XY, before 23 months of age. HR=Hazard Ratio estimate, risk of death. SE=Standard Error. Reference is XX genotype. Table summarizes results in **Fig. 2f.**

| **Time interval** | **HR** | **SE** | **P-value** | **Significance** |
| --- | --- | --- | --- | --- |
| 12mo-23mo | 0.36 | 0.60 | 0.045 | * |
| 23mo-30mo | 0.78 | 0.33 | 0.23 | n.s. |

**References**

Arnold, A. P. (2004). Sex chromosomes and brain gender. *Nature reviews. Neuroscience, 5*(9), 701-708. doi:10.1038/nrn1494

Lerman, P. M. (1980). Fitting Segmented Regression Models by Grid Search. *Journal of the Royal Statistical Society. Series C (Applied Statistics), 29*(1), 77-84. doi:10.2307/2346413

Lovell-Badge, R., & Robertson, E. (1990). XY female mice resulting from a heritable mutation in the primary testis-determining gene, Tdy. *Development, 109*(3), 635-646.

Mahadevaiah, S. K., Odorisio, T., Elliott, D. J., Rattigan, A., Szot, M., Laval, S. H., . . . Burgoyne, P. S. (1998). Mouse homologues of the human AZF candidate gene RBM are expressed in spermatogonia and spermatids, and map to a Y chromosome deletion interval associated with a high incidence of sperm abnormalities. *Human molecular genetics, 7*(4), 715-727.
